# Effect of aging and sex on cardiovascular structure and function in wildtype mice assessed with echocardiography

**DOI:** 10.1038/s41598-021-02196-0

**Published:** 2021-11-23

**Authors:** Tian Yu Zhang, Bi Jun Zhao, Tao Wang, Jia Wang

**Affiliations:** 1 The School of Basic Medicine of Air Force Medical University, Xi’ an, 710032 China; 2 Department of Cardiovascular Surgery, Rizhao Hospital Affiliated to Qingdao University, RizhaoInternational Heart Hospital, Qingdao, 276800 China; 3 Department of Medicine, University of Pennsylvania, Philadelphia, PA 19104 USA; 4 Department of Ultrasound Diagnostics, Second Affiliated Hospital of Air Force Medical University, Xi’ an, 710038 China

**Keywords:** Cardiology, Medical research

## Abstract

This study employed traditional and advanced echocardiographic techniques to assess comprehensively age- and sex-related changes in cardiovascular structure and function in wildtype (WT) mice. Forty-five normal adult wildtype mice were apportioned to groups based on age and sex: 2-month (young) male or female, and 24-month (old) male or female (*n* = 13, 13, 13, and 6, respectively). Each underwent 2-dimensional (2D) imaging echocardiography, Doppler, tissue Doppler imaging echocardiography, and speckle-tracking echocardiography (STE) for comparison of cardiovascular structure and function parameters. Compared to the young mice, the old had significantly higher body weight (BW), and lower diastolic and mean arterial pressure. The left ventricular (LV) end-diastolic and end-systolic volumes, and left ventricular mass, were significantly higher in the old mice. Within each sex, the cardiac diastolic and systolic function parameters were comparable between the young and old. Isovolumetric relaxation time (IVRT)/diastolic time interval (DT) and the maximum drop rate of pressure in LV (− dP/dtmax) were significantly lower in the old mice, while the LV relaxation time constant (Tau) was significantly higher. Spearman’s rank correlation showed a positive association between IVRT/DT and − dp/dtmax (male *r* = 0.663; female *r* = 0.639). Among the males, the maximum rise rate of pressure in LV (+ dp/dtmax), and systolic global longitudinal strains and rates (S-GLS, S-GLSR) were significantly different between the young and old. Spearman’s rank correlation showed positive association between S-GLS, S-GLSR and + dp/dtmax (r = 0.709 and r = 0.499). Regarding vascular structure, the ascending aorta systolic and diastolic diameters were significantly higher in the old mice compared with the young. The male mice had progressive, age-related aortic stiffness. Ageing in mice leads to changes in cardiovascular structure and cardiac diastolic function, but systolic function is relatively well preserved in females. Changes in cardiac function and arterial stiffness were more significant in males than females. Traditional ECG is better than STE for evaluating LV diastolic function; STE is better for LV systolic function.

## Introduction

In humans, the heart and blood vessels undergo changes in structure and function (SF) with advancing age that lead to the development of various diseases^1^. Epidemiological studies^2–4^ have shown that arterial stiffness and hypertension, and related cardiovascular diseases such as stroke and myocardial infarction, are more prevalent in the aged than the young. Strait et al.^5^ showed that impairment of cardiac diastolic function occurs with aging, but systolic function is preserved.

Age-associated changes in cardiovascular SF may be monitored using the current echocardiographic (ECG) techniques. Mice have a naturally short lifespan, which makes the mouse a particularly interesting model for longitudinal studies. Mice age more quickly than humans do, and a mouse at 24 months of age is equivalent to a human in his seventh decade, according to the probability of survival curves generated by the National Institutes on Aging^6^.

Recent advances in imaging technology, with improved resolution, have made ECG in mice and rats a valuable and generally well-accepted tool for understanding the physiological and pathophysiological changes associated with ageing and cardiovascular diseases^7^. Yet, there is little comprehensive study about age- and sex-related changes in mice with regard to cardiovascular SF. The present study employed traditional echocardiography and advanced ECG techniques, comparing with gold standard for evaluating cardiovascular function, to assess comprehensively the age- and sex-related changes in cardiovascular SF in WT mice.

## Methods

### Experimental mice

Forty-five normal adult WT mice were apportioned into 4 groups according to age and sex: 2-month-old male (*n* = 13); 24-month-old male (*n* = 13); 2-month-old female (*n* = 13); and 24-month-old female (*n* = 6). The 2- and 24-month-old mice were considered young and old, respectively.

All mice were bred and maintained at the Model Animal Research Center and maintained in the Animal Laboratory Resource Facility, both at Pennsylvania University. All the experiments were performed in compliance with the Guide for the Care and Use of Laboratory Animals published by the United States National Institutes of Health (NIH Publication, 8th Edition, 2011). The animal care and experimental protocols were approved by the University of Pennsylvania Committee on Animal Care. The study was carried out in compliance with the ARRIVE guidelines.

### Standard ECG measurements

A high-frequency ultrasound device (Vevo 2100, VisualSonics, Toronto, Canada) was used for ultrasound imaging. We used two types of linear array probes, MS-250S (frequency 13–24 MHz) and MS-550D (frequency 22–55 MHz). The MS-250S probe was mainly used to measure the heart function of mice, and MS-550D was mainly focused on measure the vascular function of mice. All mice were measured for BW before the operation. During the procedure, the mice were anesthetized with 2–3% isoflurane in an induction chamber and maintained with 1.0–1.5% isoflurane delivered via 100% O_2_ mask inhalation. After shaving and removing the hair on the neck, chest, and abdomen with depilatory cream, the mice were placed supine on a controlled heating pad. The body temperature was maintained at 37 °C and electrocardiogram limb electrodes were placed. The heart rate (HR), respiration frequency, and body temperature were continuously monitored. All ECG measurements were the average of 3 cardiac cycles. The acquired data are detailed below.

### 2D imaging ECG measurements

**Figure 1:**
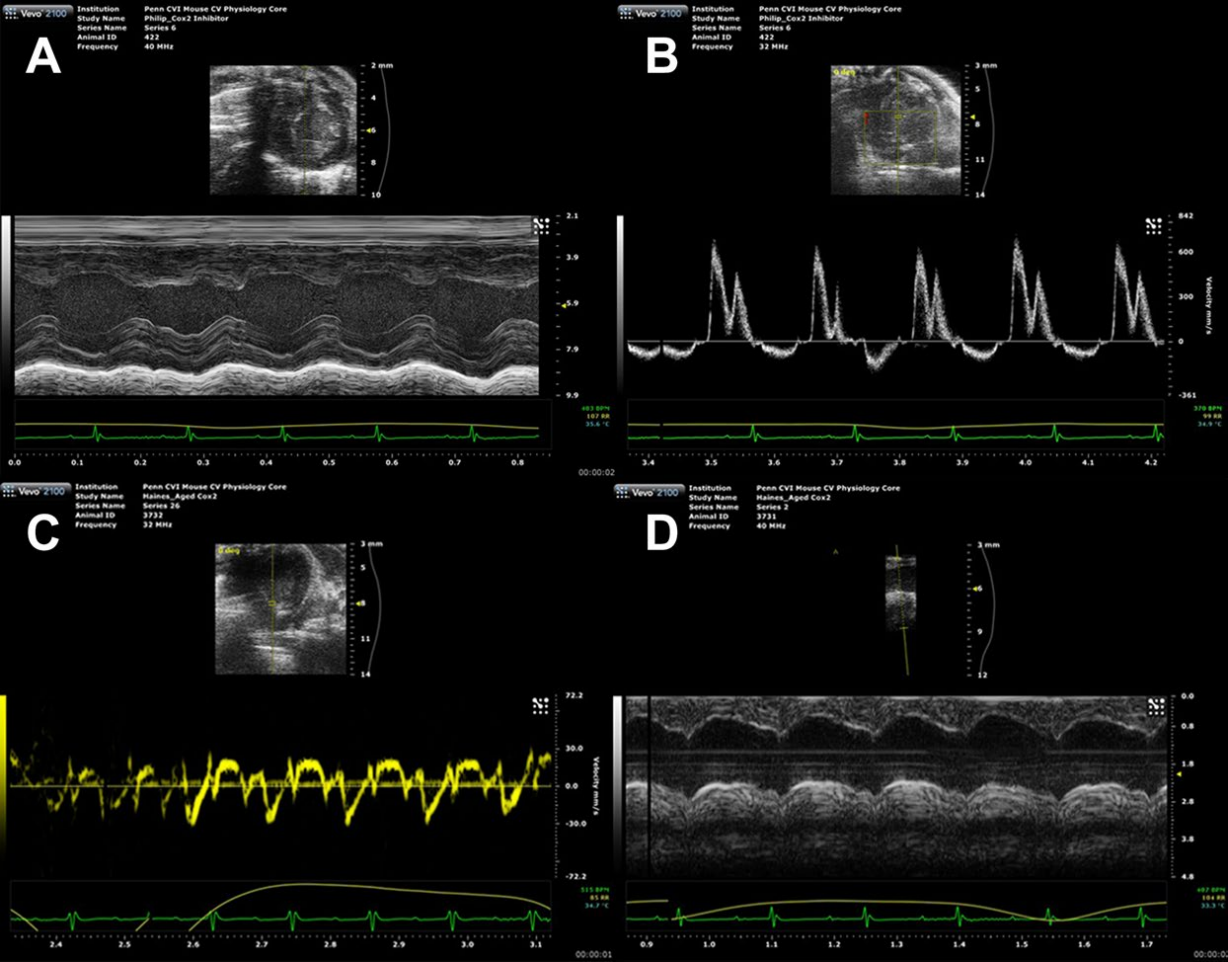
ECG 2D and Doppler imaging measurements. (**A**) M-mode imaging of the LV mid-papillary level in the parasternal short-axis view. (**B**) Mitral valve inflow velocities were recorded by Doppler ECG in the apical 4-chamber view. (**C**) LV myocardial velocities were recorded by TDI in the apical 4-chamber view. (**D**) AADs and AADd were measured in M-mode imaging. *AADs ascending aorta diameters at end-systole; A*ADd* ascending aorta diameters at end-diastole.

The following were measured from the LV mid-papillary level in the parasternal short-axis view with 2D M-mode imaging (Fig. 1A): interventricular septum thicknesses at end-diastole and end-systole (IVSd and IVSs, respectively); LV internal diameters at end-diastole and end-systole (LVIDd, LVIDs); LV posterior wall thicknesses at end-diastole and end-systole (LVPWd, LVPWs); and stroke volume (SV), which was derived by the formula: EDV-ESV, where EDV and ESV are the end-diastolic and end-systolic volumes. The LV systolic function was estimated by the ejection fraction (EF), which was derived by the formula: SV/EDV. The area-length method^8^ was used to calculate LV mass and relative wall thickness (RWT), which were derived using the following formulas: LV mass, g = 1.053([LVIDd + IVSd + LVPWd]3 − [LVIDd]^3^), and RWT = (IVSd + LVPWd)/LVIDd.

### Doppler and tissue Doppler imaging ECG measurements

Doppler measurements of the mitral valve inflow velocities were recorded from an apical 4-chamber view, with a cursor positioned by the tips of the mitral valve leaflets. Diastolic function was evaluated from measurements of the early filling velocity (E) and the atrial filling velocity (A), and calculation of the E/A ratio. The following values were measured using mitral valve spectral Doppler (Fig. 1B): isovolumetric relaxation time (IVRT), isovolumetric contraction time (IVCT), mitral valve ejection time (MVET), and aortic ejection time (AET). Systolic time interval (ST = IVCT + AET), diastolic time interval (DT = IVRT + MVET), IVRT/DT, IVCT/ST, and myocardial performance index (MPI) were calculated with the formula: (IVRT + IVCT)/AET. MPI is a useful index to assess cardiac systolic and diastolic function in mice. Tissue Doppler imaging (TDI) was used to evaluate LV myocardial velocities. The pulsed TDI sample volume was determined at the level of the septal mitral valve annulus in the apical 4-chamber view. Measurement of the early diastolic (e) and late diastolic (a) velocities and calculation of e/a, E/e ratios were performed (Fig. 1C).

### Speckle tracking echocardiography (STE) measurements

Speckle tracking-based strain analysis of 3 consecutive cardiac cycles 2D gray scale ECG images were acquired from the parasternal long- and short-axes using the Vevostrain software package (VisualSonics). Semi-automated tracings were performed on the endocardial and epicardial borders, which were then corrected as needed to achieve good quality tracking throughout each cine loop. Strain measurements were performed by processing the tracked images in a frame-by-frame manner, and were averaged from the acquired cardiac cycles, from which curvilinear strain and strain rate data were obtained. Each of the LV myocardium long- and short-axis views was divided by Vevo Software into 6 standard anatomic segments.

**Figure 2:**
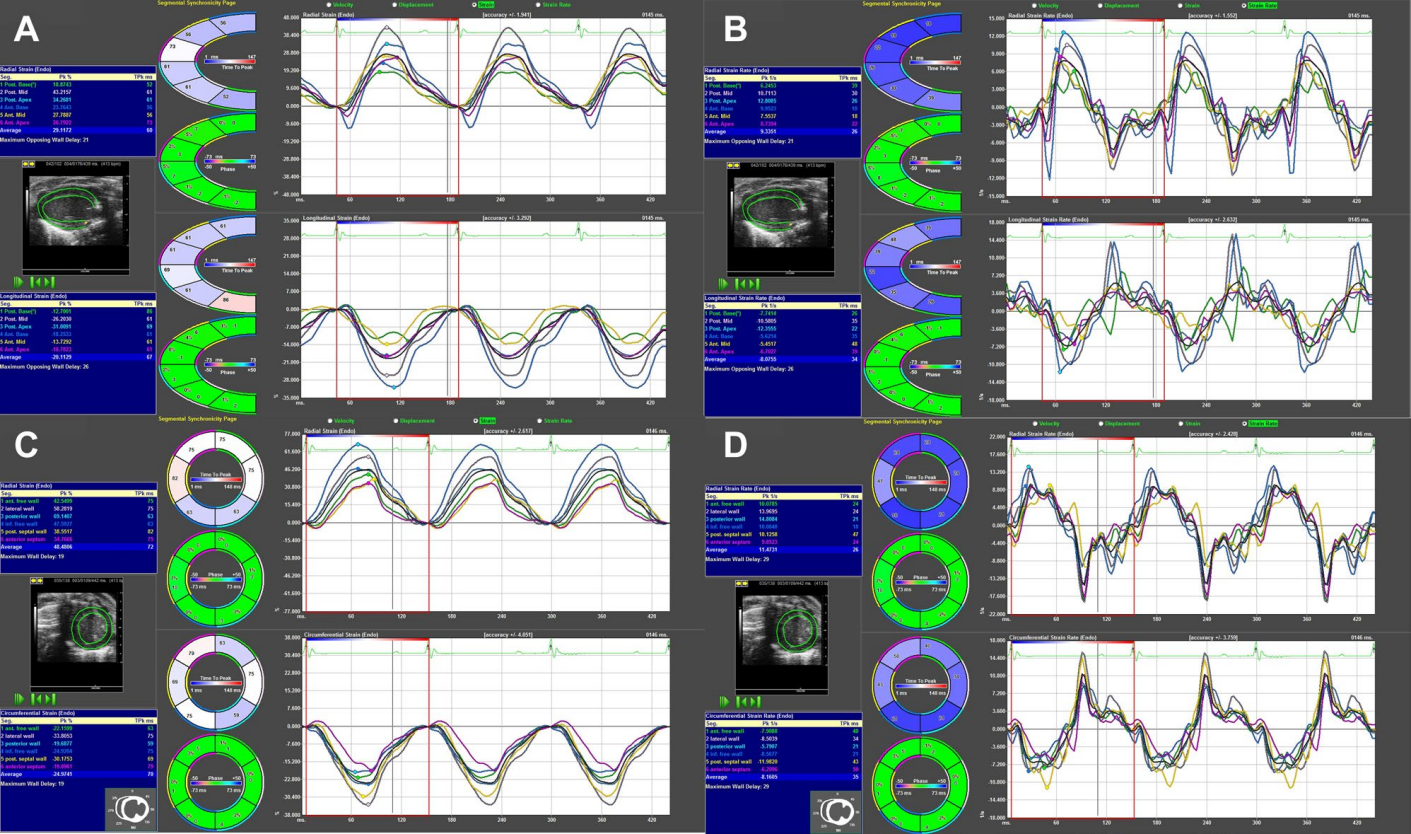
Global and regional strain and strain rate analyses in parasternal long-axis and short-axis view. (**A**) GLS and RLS analyses in parasternal long-axis view. (**B**) GLSR and RLSR analyses in parasternal long-axis view. (**C**) GCS, RCS, GRS, and RRS analyses in parasternal short-axis view. (**D**) GCSR, GRSR, RCSR, RRSR analyses in parasternal short-axis view. *GLS global longitudinal strain; *GLSR* global longitudinal strain rate; *RLS* regional longitudinal strain; *RLSR* regional longitudinal strain rate; *GCS* global circumferential strain; *GCSR* global circumferential strain rate; *RCS* regional circumferential strain; *RCSR* regional circumferential strain rate; *GRS* global radial strain; *GRSR* global radial strain rate; *RRS* regional radial strain; *RRSR* regional radial strain rate.

Analyses of the following were performed from parasternal long-axis views (Fig. 2A,B): systolic global and regional longitudinal strain (S-GLS, S-RLS ), strain rate (S-GLSR, S-RLSR), and diastolic global longitudinal strain rate (D-GLSR). From parasternal short-axis views (at the mid-papillary level; Fig. 2C,D) analyses of the following were gained: systolic global and regional radial strain (S-GRS, S-RRS), radial strain rate (S-GRSR, S-RRSR), circumferential strain(S-GCS, S-RCS), and circumferential strain rates (S-GCSR, S-RCSR), and diastolic global radial and circumferential strain rate (D-GRSR, D-GCSR). All images were acquired at a frame rate of > 200 frames/s (average, 230 frames/s) and at an average depth of 11 mm. All strain analyses were performed by a single trained investigator.

### Aortic diameter and pulse wave velocity measurements

The probe was placed vertically in the mouse neck, slightly left, to show clearly the ascending aortic lumen and aortic valve (AV) by adjusting the position of the probe. The ascending aorta diameters at end-systole and end-diastole (AADs and AADd, respectively) were measured in M-mode imaging (Fig. 1D). The rate of aorta diameter change rate (∆AADR) was calculated using the formula: (AADs–AADd)/AADs. The ascending aorta and abdominal aorta (the iliac bifurcation) were selected for pulsed Doppler image acquisition. The sample gate of the pulse wave was made as small as possible with adjusted positions to obtain the clearest pulse waveforms.

The distance between the carotid and iliac arteries was measured in millimeters with Vernier calipers, defined as the difference between the distance from the iliac to the nose hood, and the distance from the ascending aorta to the nose hood. The pulse-transit time from the ascending aorta to the iliac arteries was obtained by subtracting the time intervals of the mean right-ascending aorta from the mean right-iliac foot. The pulse wave velocity (PWV) was calculated as the distance between the carotid and iliac arteries divided by the pulse-transit time.

### Invasive cardiac catheter measurements

Invasive cardiac catheter measurements of cardiovascular function were performed at the end of study. Under anesthesia with tribromoethanol (125–300 mg/kg intraperitoneal), and/or 1–2% isoflurane, each mouse was placed on a heating pad to maintain body temperature. The responsiveness of the mouse was tested by toe pinching. If the animal responded, additional anesthesia was administered. The surgical areas were shaved and antiseptic agents (betadine and 70% ethanol) were applied. The mouse was secured to the operating field with surgical tape. A blunt dissection was performed over the thin muscle layer around the throat, and the right carotid artery was exposed and isolated. The suture around the distal end of the artery was secured. A suture was then placed loosely around the proximal end of the artery, on which a small metal hemostat clip was placed to minimize bleeding during catheter insertion. After making a tiny incision near the distal end of the artery with micro-scissors, a pressure or pressure–volume loop catheter (1F or 1.4F) was gently inserted into the artery while the catheter tip was quickly advanced down the ascending aorta via the aortic valve into the left ventricle. The proximal suture was tightened to minimize blood loss.

The pressure or pressure–volume loop data was collected and cardiovascular intervention (vena cava occlusion, drug injection) was performed. To measure the pressure gradient over the aortic-banded region, another pressure catheter was inserted via the abdominal artery or femoral artery. Before and after isoproterenol (or dobutamine) dosing (intravenous or intraperitoneal), the following hemodynamic data were recorded: The peak systolic and diastolic pressures (SP, DP); mean arterial pressure (MAP); Tau; + dp/dtmax and − dp/dtmax.

### Statistical analysis

All echo data were analyzed offline by a single trained observer. SPSS 20.0 (SPSS, Chicago, IL, USA) was used for all analyses. All measurements are shown as mean ± standard error of at least 3 independent assays, unless otherwise noted. The independent samples *t*-test was used to compare the data between groups of the same sex. Spearman’s rank correlation test was used to assess a correlation between parameters. Probability (*P*) values < 0.05 and < 0.01 were considered significant and very significant, respectively.

## Results

### Basic parameters

Compared with the young mice, the BW of the old mice were significantly higher, and DP and MAP were significantly lower (*P* < 0.01). Within sex, the young and old mice were comparable with regard to HR. The SP of the old females was very significantly lower than that of young females (*P* < 0.01), but between the young and old males the SP were comparable (Table 1).

**Table 1:** Basic parameters of the study population.

	Male			Female		
	Young	Old	*P*^a^	Young	Old	*P*^b^
Sample size, *n*	13	13	–	13	6	–
BW, g	26.33 ± 0.44	33.18 ± 0.87	0.000	21.47 ± 0.03	28.28 ± 1.17	0.000
HR, bpm	528.15 ± 13.15	494.77 ± 10.68	0.054	538.77 ± 14.84	544.17 ± 8.66	0.816
SP, mmHg	94.18 ± 2.68	78.51 ± 1.86	0.257	99.73 ± 4.50	71.87 ± 3.48	0.002
DP, mmHg	65.95 ± 2.33	48.89 ± 1.69	0.000	69.37 ± 4.77	43.58 ± 2.41	0.002
MAP, mmHg	80.83 ± 2.42	63.12 ± 1.69	0.000	84.74 ± 4.61	56.98 ± 3.13	0.001

^a^Between young and old males; ^b^between young and old females.

*BW* body weight; *HR* heart rate; *SP* systolic pressure; *DP* diastolic pressure; *MAP* mean arterial pressure.

### Cardiac structural parameters

Compared with the young mice, the cardiac structural parameters LV mass, EDV and ESV of the old mice were significantly higher (*P* < 0.05). Between the young and old females, the LV mass/BW, RWT, and SV of the old females were significantly higher than that of the young females (*P* < 0.05), but between the young and old males, these parameters were comparable (Table 2).

**Table 2:** Cardiac structural ECG parameters of the study population.

	Male			Female		
	Young	Old	*P*^a^	Young	Old	*P*^b^
Sample size, *n*	13	13	–	13	6	–
LV mass, mg	76.84 ± 1.16	93.86 ± 2.34	0.000	61.07 ± 1.15	91.69 ± 5.69	0.000
LV mass/BW, mg/g	2.93 ± 0.06	2.85 ± 0.10	0.489	2.84 ± 0.04	3.24 ± 0.15	0.003
RWT	0.42 ± 0.01	0.46 ± 0.01	0.084	0.45 ± 0.02	0.51 ± 0.03	0.047
EDV, µL	61.18 ± 2.46	72.21 ± 4.08	0.028	47.10 ± 1.88	66.85 ± 3.18	0.000
ESV, µL	26.15 ± 1.23	32.24 ± 2.61	0.042	21.22 ± 0.96	30.49 ± 1.31	0.000
SV, µL	35.04 ± 1.84	39.97 ± 2.17	0.106	25.88 ± 1.30	36.37 ± 1.97	0.000

^a^Between young and old males; ^b^between young and old females.

*BW* body weight; *RWT* relative wall thickness; *EDV* end-diastolic volume; *ESV* end-systolic volume; *SV* stroke volume.

### Cardiac diastolic functional parameters

**Figure 3:**
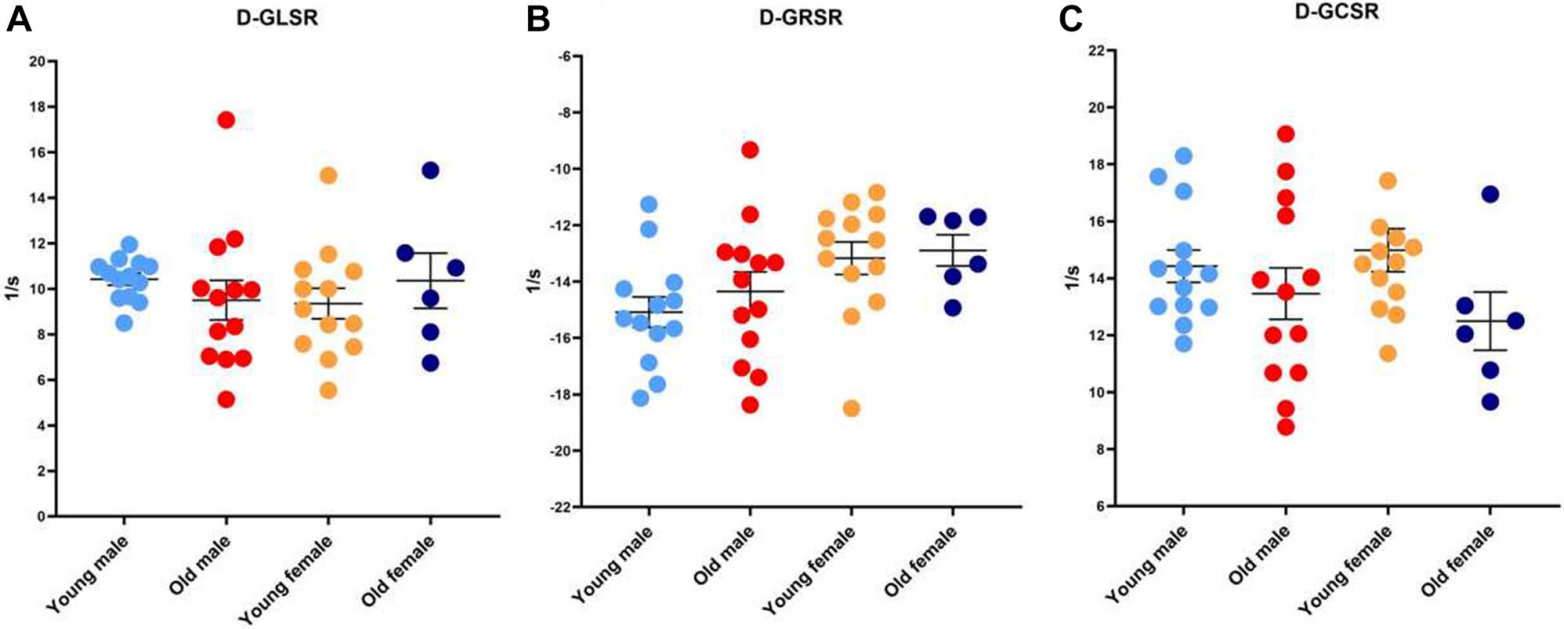
Cardiac diastolic functional stain parameters. D-GLSR parameters (**B**) D-GRSR parameters (**C**) D-GCSR parameters. *D-GLSR diastolic global longitudinal strain rate; *D-GRSR* diastolic global radial strain rate; *D-GCSR* diastolic global circumferential strain rate.

**Figure 4:**
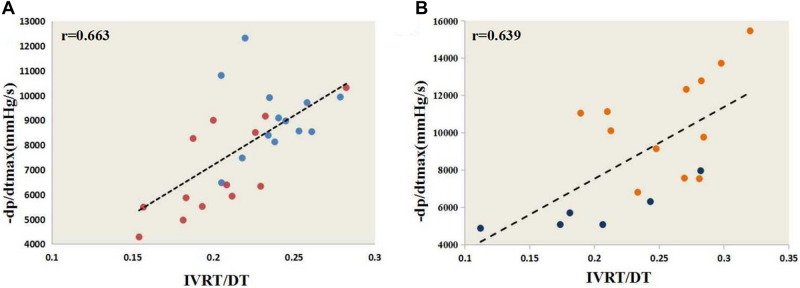
The correlation of IVRT/DT with − dP/dtmax. (**A**) IVRT/DT and − dp/dtmax correlation analyses in males by Spearman’s rank correlation test. (**B**) IVRT/DT and − dp/dtmax correlation analyses in females by Spearman’s rank correlation. * − *dp*/*dtmax* the maximum drop rate of pressure in left ventricle; *IVRT/DT* isovolumetric relaxation time/ diastolic time interval.

Compared with the young mice, the cardiac diastolic functional parameters IVRT/DT and − dP/dtmax were significantly lower in the old mice, while Tau was significantly higher in the old mice. Within each sex, the young and old mice were similar with regard to each of the following diastolic functional values: E, E/e ratios, DT, and D-GLSR, D-GRSR, and D-GCSR. The e and e/a ratios of the young and old females were similar, but that of the old males was significantly lower than that of the young males. The IVRT of the young and old males were similar, but that of the old females was significantly lower than that of the young females (Table 3; Fig. 3). Spearman’s rank correlation test indicated a significant positive correlation between IVRT/DT and − dp/dtmax (male r = 0.663; female r = 0.639)(Fig. 4). There were no significant correlation between − dP/dtmax and diastolic strain parameters except for D-GCSR in females (r = 0.577) (Table 4).

**Table 3:** Cardiac diastolic functional parameters of the study population.

	Male			Female		
	Young	Old	*P*^a^	Young	Old	*P*^b^
Sample size, *n*	13	13	–	13	6	–
E, mm/s	778.32 ± 19.68	737.16 ± 35.11	0.325	763.19 ± 31.78	703.57 ± 31.25	0.265
e, mm/s	17.12 ± 2.62	9.59 ± 1.45	0.015	8.26 ± 1.64	13.91 ± 3.95	0.131
E/e	107.66 ± 39.74	115.98 ± 30.36	0.833	152.37 ± 31.17	97.85 ± 37.05	0.314
e/a	0.70 ± 0.12	0.40 ± 0.07	0.027	0.39 ± 0.11	0.50 ± 0.15	0.596
IVRT, ms	14.90 ± 0.49	13.70 ± 0.71	0.244	16.04 ± 0.74	12.32 ± 1.56	0.024
DT, ms	63.04 ± 2.57	68.59 ± 2.36	0.137	62.85 ± 3.51	61.90 ± 2.37	0.862
IVRT/DT	0.24 ± 0.01	0.20 ± 0.01	0.003	0.26 ± 0.01	0.20 ± 0.02	0.017
D-GLSR	10.42 ± 0.26	9.50 ± 0.87	0.394	9.35 ± 0.67	10.36 ± 1.21	0.444
D-GRSR	–15.09 ± 0.54	–14.35 ± 0.70	0.436	–12.89 ± 0.55	–12.89 ± 0.55	0.769
D-GCSR	14.42 ± 0.57	13.46 ± 0.90	0.360	14.99 ± 0.76	12.50 ± 1.02	0.075
Tau, s	0.010 ± 0.034	0.012 ± 0.002	0.003	0.009 ± 0.000	0.011 ± 0.000	0.006
− dP/dtmax, mmHg/s	9568.41 ± 479.60	6930.81 ± 523.21	0.047	10,624.24 ± 766.77	5844.365 ± 476.88	0.000

^a^Between young and old males; ^b^between young and old females.

*E* early filling velocity, *e* early diastolic velocity, *a*: late diastolic velocity, *IVRT*: isovolumetric relaxation time, *DT* diastolic time interval, *D-GLSR* diastolic global longitudinal strain rate, *D-GRSR* diastolic global radial strain rate, *D-GCSR*: diastolic global circumferential strain rate, *Tau*: LV relaxation time constant, − *dp*/*dtmax* the maximum drop rate of pressure in left ventricle.

**Table 4:** The correlation of − dP/dtmax with diastolic strain parameters.

	D-GLSR	D-GRSR	D-GCSR
Male	0.180	0.220	0.160
Female	0.202	0.104	0.577

*D-GLSR* diastolic global longitudinal strain rate, *D-GRSR* diastolic global radial strain rate, *D-GCSR* diastolic global circumferential strain rate.

### Cardiac systolic functional parameters

**Figure 5:**
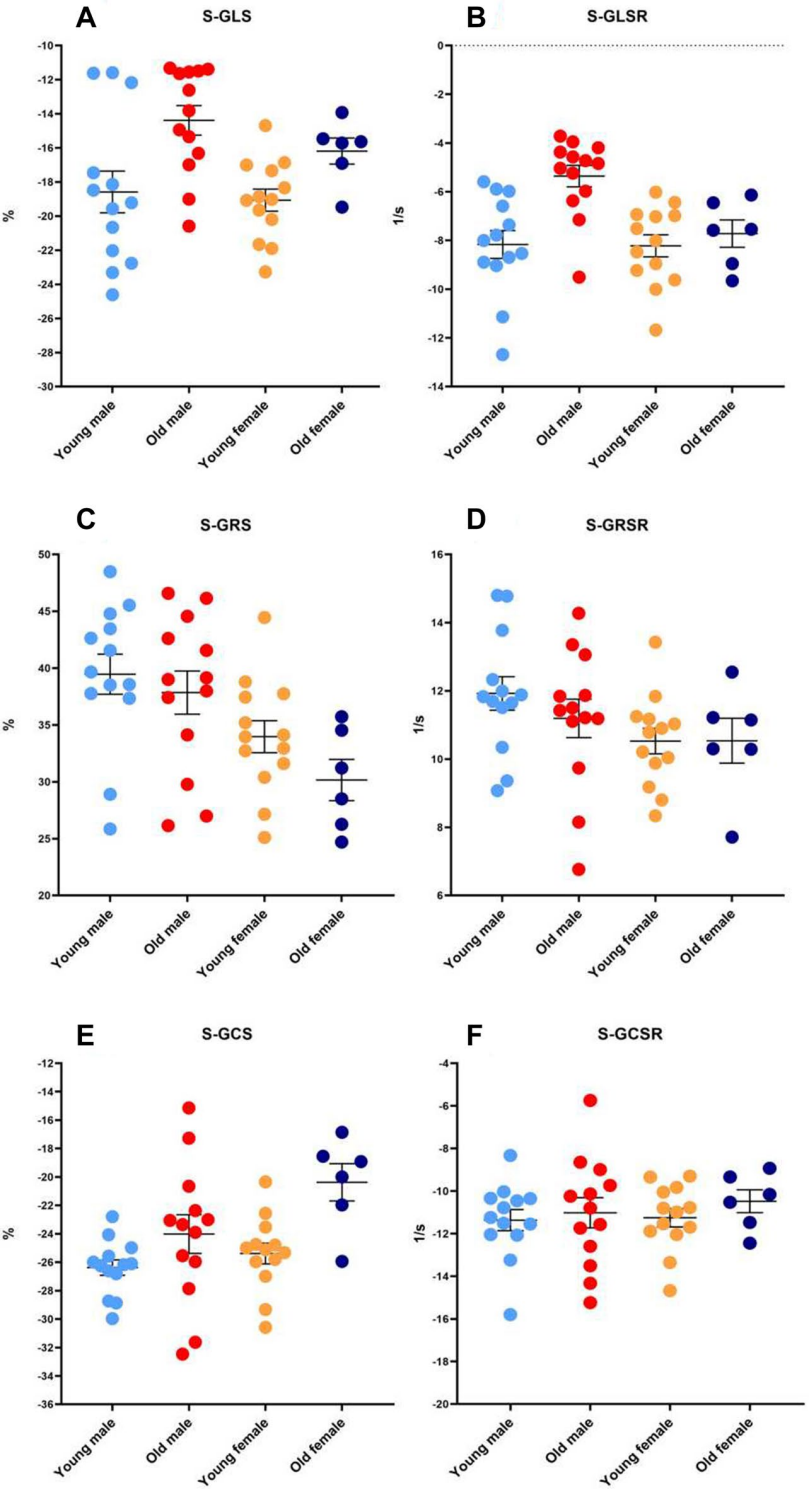
Cardiac systolic functional global strain parameters. (**A**) systolic global longitudinal strain (**B**) systolic global longitudinal strain rate (**C**) systolic global radial strain (**D**) systolic global radial strain rate (**E**) systolic global circumferential strain (**F**) systolic global circumferential strain rate. *S-GLS systolic global longitudinal strain; *S-GLSR* systolic global longitudinal strain rate; *S-GRS* systolic global radial strain; *S-GRSR* systolic global radial strain rate; *S-GCS* systolic global circumferential strain; *S-GCSR* systolic global circumferential strain rate.

**Figure 6:**
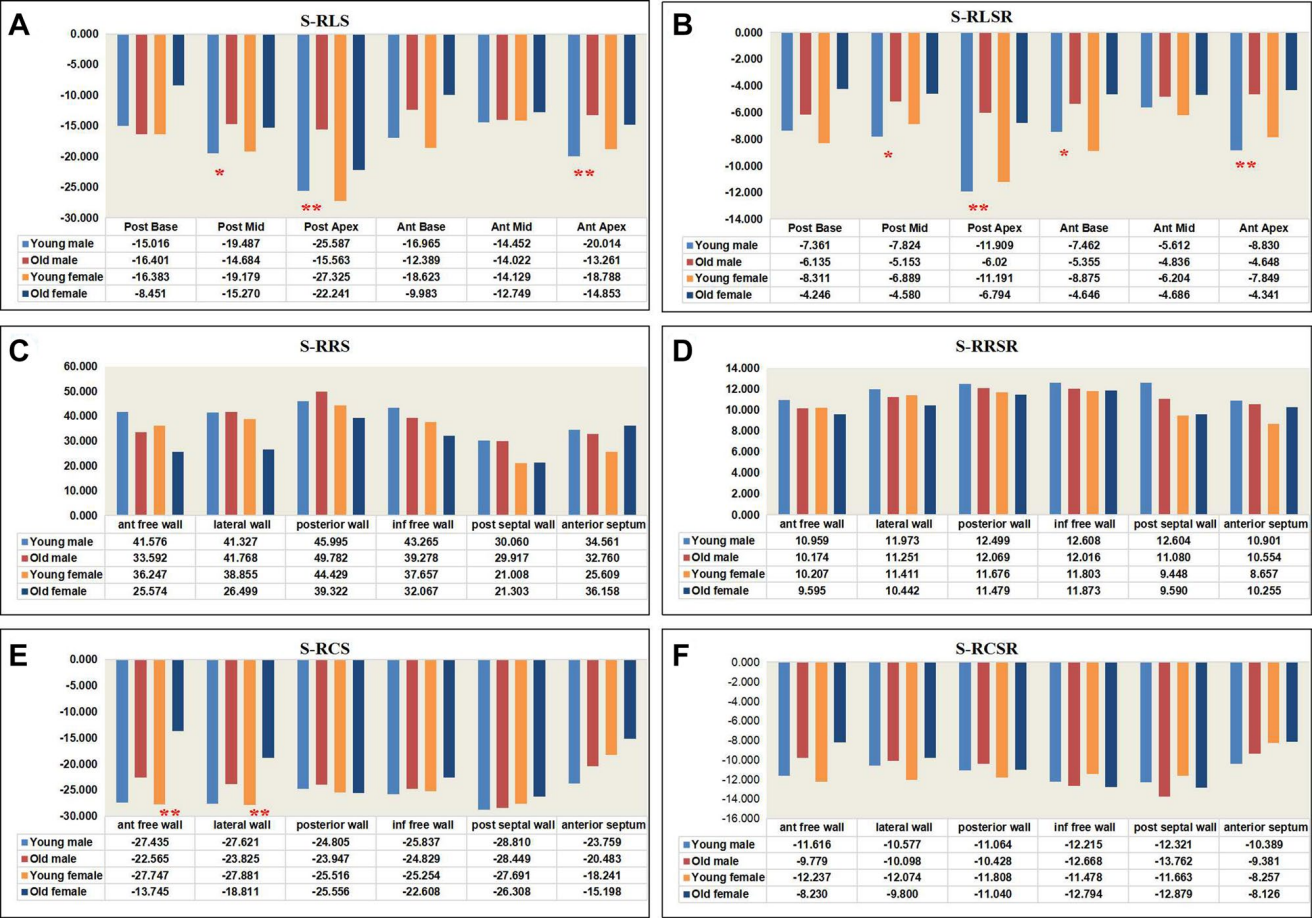
Cardiac systolic functional regional strain parameters. (**A**) systolic regional longitudinal strain (**B**) systolic regional longitudinal strain rate (**C**) systolic regional radial strain (**D**) systolic regional radial strain rate (**E**) systolic regional circumferential strain (**F**) systolic regional circumferential strain rate. *S-RLS systolic regional longitudinal strain; *S-RLSR* systolic regional longitudinal strain rate; *S-RRS* systolic regional radial strain; *S-RRSR* systolic regional radial strain rate; *S-RCS* systolic regional circumferential strain; *S-RCSR* systolic regional circumferential strain rate. Old males versus young males *P < 0.05, **P < 0.01. Old females versus young females ^#^*P* < 0.05, ^##^*P* < 0.01.

Within each sex, each of the following cardiac systolic functional parameters were similar between the young and old groups: EF, IVCT, ST, IVCT/ST, MPI, S-GRS, S-GRSR, S-GCSR, S-RRS, S-RRSR and S-RCSR (Table 5; Figs. 5C,D,F and 6C,D,F).

**Table 5:** Cardiac systolic functional parameters of the study population.

	Male			Female		
	Young	Old	*P*^a^	Young	Old	*P*^b^
Sample size, *n*	13	13	–	13	6	–
EF	0.57 ± 0.02	0.56 ± 0.02	0.578	0.55 ± 0.01	0.54 ± 0.01	0.799
IVCT, ms	13.44 ± 0.85	14.71 ± 1.01	0.384	12.21 ± 0.42	12.19 ± 1.38	0.987
ST, ms	58.69 ± 2.04	59.81 ± 2.19	0.732	58.43 ± 1.40	55.10 ± 1.73	0.179
IVCT/ST	0.23 ± 0.01	0.24 ± 0.01	0.287	0.21 ± 0.01	0.22 ± 0.02	0.629
MPI	0.63 ± 0.02	0.64 ± 0.01	0.987	0.63 ± 0.02	0.58 ± 0.04	0.235
S-GLS	− 18.59 ± 1.22	− 14.39 ± 0.87	0.005	− 19.07 ± 1.57	− 16.19 ± 0.80	0.085
S-GLSR	− 8.17 ± 0.94	− 5.36 ± 0.44	0.007	− 8.22 ± 0.78	− 7.72 ± 0.36	0.514
S-GRS	39.46 ± 1.76	37.85 ± 1.90	0.496	33.97 ± 1.61	30.15 ± 2.45	0.206
S-GRSR	11.92 ± 0.49	11.19 ± 0.56	0.274	10.53 ± 0.38	10.54 ± 0.33	0.993
S-GCS	− 26.38 ± 0.54	− 24.02 ± 1.37	0.085	− 25.39 ± 0.73	− 20.37 ± 0.95	0.000
S-GCSR	− 11.36 ± 0.50	− 11.02 ± 0.71	0.586	− 11.25 ± 0.44	− 10.48 ± 0.57	0.318
+ dP/dtmax, mmHg/s	9873.15 ± 868.93	6492.26 ± 370.90	0.004	8843.45 ± 843.45	7815.55 ± 815.55	0.284

^a^Between young and old males; ^b^between young and old females.

*EF* ejection fraction, *IVCT* isovolumetric contraction time, *ST* systolic time interval, *MPI* myocardial performance index, *S-GLS* systolic global longitudinal strain, *S-GLSR* systolic global longitudinal strain rate, *S-GRS* systolic global radial strain, *S-GRSR* systolic global radial strain rate, *S-GCS* systolic global circumferential strain, *S-GCSR* systolic global circumferential strain rate, + *dp*/*dtmax* the maximum rise rate of pressure in left ventricle.

**Figure 7:**
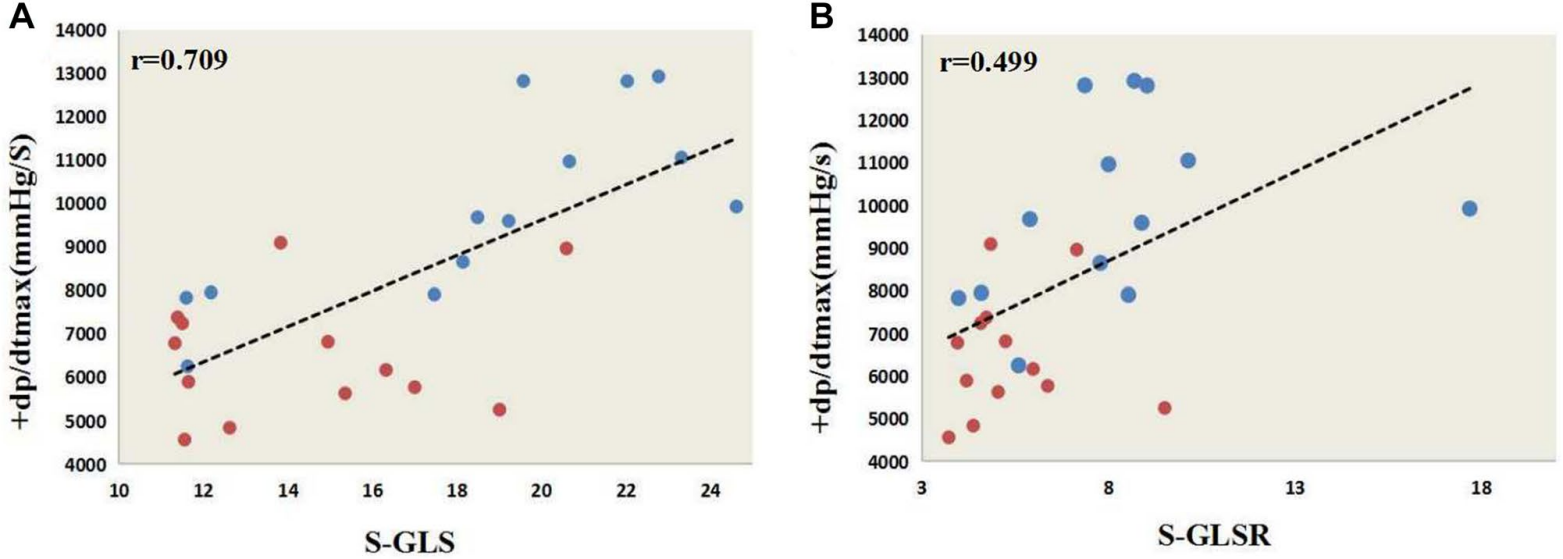
The significant correlation of + dP/dt with systolic functional parameters. (**A**) S-GLS and + dp/dtmax correlation analyses in males by Spearman’s rank correlation. (**B**) S-GLSR and + dp/dtmax correlation analyses in males by Spearman’s rank correlation. * + dp/dtmax the maximum rise rate of pressure in left ventricle; S-GLS systolic global longitudinal strain; *S-GLSR* systolic global longitudinal strain rate.

The + dp/dtmax, S-GLS, S-GLSR of the young and old females were similar. Among the males, the + dp/dtmax of and S-GLS and S-GLSR the old males was significantly lower than that of the young males (Table 5; Fig. 5A,B). S-RLS and S-RLSR showed obvious difference mainly in anterior apex and posterior apex (Fig. 6A,B). The S-GCS of the young and old males were similar, but that of the old females was significantly lower than that of the young females (Table 5; Fig. 5E), S-RCS showed obvious difference mainly in anterior free wall and lateral wall (Fig. 6E). There were no significant correlation between + dP/dtmax and systolic strain parameters except for S-GLS, S-GLSR in males (r = 0.709 and r = 0.499). (Table 6; Fig. 7).

**Table 6:** The correlation of + dP/dtmax with systolic strain parameters.

	S-GLS	S-GLSR	S-GRS	S-GRSR	S-GCS	S-GCSR
Male	0.709	0.499	0.117	0.152	0.104	0.271
Female	0.412	0.348	0.365	0.162	0.395	0.076

*S-GLS* systolic global longitudinal strain, *S-GLSR* systolic global longitudinal strain rate, *S-GRS* systolic global radial strain, *S-GRSR* systolic global radial strain rate, *S-GCS* systolic global circumferential strain, *S-GCSR* systolic global circumferential strain rate.

### Vascular SF parameters

**Figure 8:**
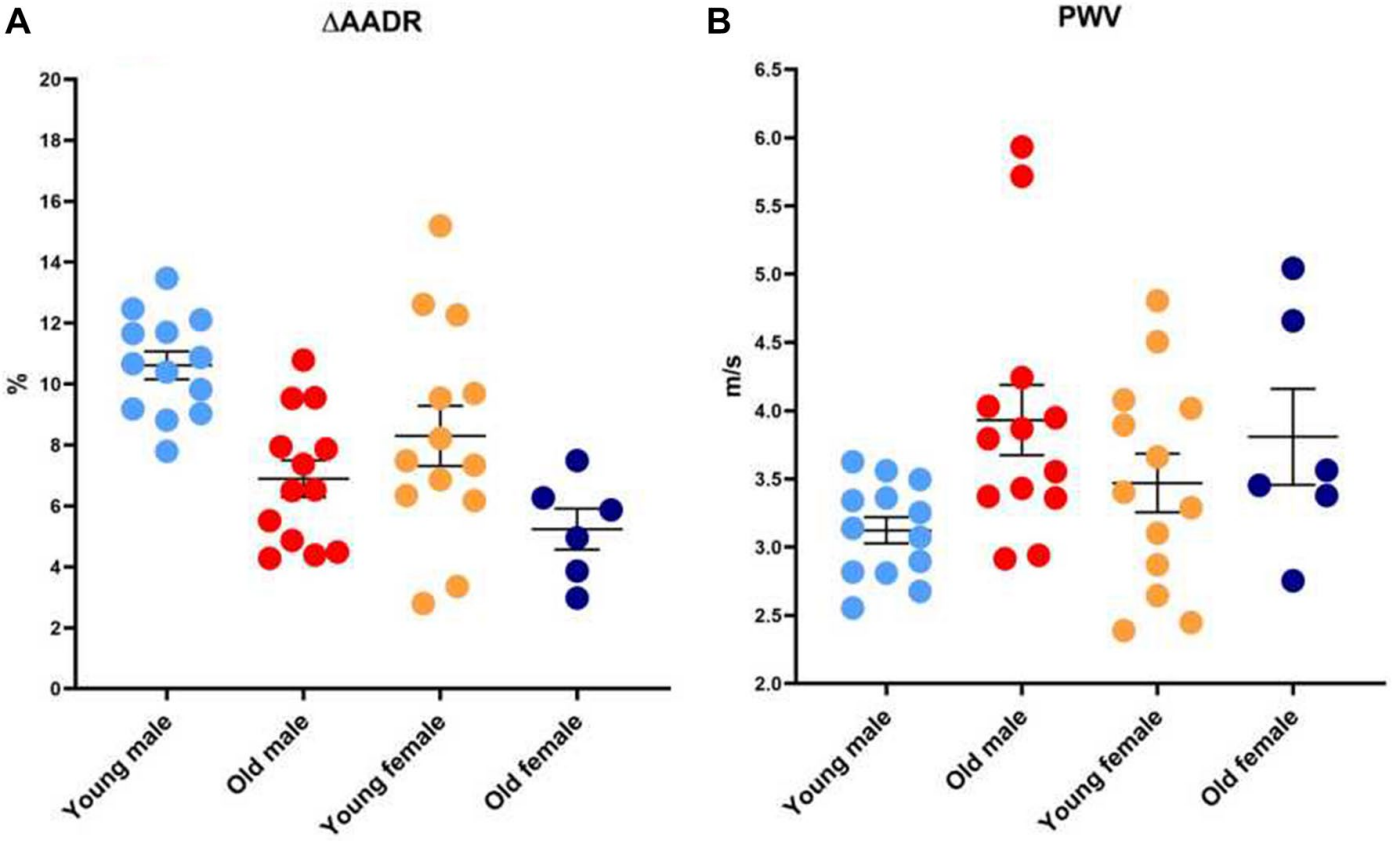
Vascular SF parameters. (**A**) Comparison of ∆AADR between old and young groups. (**B**) Comparison of PWV between old and young groups.

Regarding the vascular structural parameters, the ascending aorta systolic and diastolic diameters (AADs and AADd) of the old groups were significantly higher compared with that of the young. Concerning vascular functional parameters, compared with the young males, the ∆AADR of the old males were significantly lower, and the PWV were significantly higher. Between the female groups, the ∆AADR and PWV were similar (Fig. 8).

## Discussion

The process of aging is evolutionarily conserved and yet poorly understood. Cardiovascular disease accounts for more than 30% of global deaths, and heart failure is the leading cause of death for persons older than 65 years. The age-associated changes in cardiovascular SF may well be reflected by the occurrence, severity, and prognosis of cardiovascular disease^9^. Murine animals are very similar to humans in terms of genome, cardiovascular anatomy, and physiology^10^. STE has been widely used in clinical cardiovascular disease, and is a quantitative, rapid, and accurate method to evaluate left and right ventricular function^11,12^. Greenberg et al.^13^ reported the mean rate of strain as an index to evaluate LV systolic function. De Lucia and co-workers^14^ are one of the groups that have published age-related changes in cardiac systolic and diastolic function using conventional ECG and STE in a mouse model. They reported that, compared with conventional ECG, STE reflects subtle changes in LV global/regional strain, relaxation, and synchronicity at earlier stages of aging. In the present study, we not only used conventional ECG parameters, but also STE to evaluate LV diastolic and systolic function in mice. Thus, we obtained data for a wide range of parameters and evaluated changes in heart function more comprehensively and accurately.

Normal cardiac aging is characterized by changes in SF. In conventional ECG, the evaluation of LV diastolic function employs the E, A, E/A ratio, IVRT, DT, IVRT/DT and TDI values (e, a, e/a and E/e ratio)^15^. In the present study, because of the fast heart rate of mice, we could not readily identify the E and A in most of these animals, and therefore only recorded the E. We found that 24-month-old mice exhibited obvious structural changes and modest declines in diastolic function compared with the 2-month-old mice. Important factors that contribute to abnormal diastolic function with normal aging include greater cardiomyocyte size, higher rates of apoptosis with lower myocyte numbers, increased collagen deposition, and functional changes at the cellular level^16^. These changes may result in greater LV diastolic stiffness with aging^17^. At present, the gold standard for evaluating LV diastolic function is the constant Tau and − dP/dtmax, which are measured by cardiac catheterization^18^. Compared with the gold standard, we found that IVRT/DT was the most sensitive parameter to reflect LV diastolic function, as determined by ECG. STE was no better than the conventional ECG for evaluating LV diastolic function in mice. Our study also found that the e/a ratio was significantly lower in the old male mice, relative to the young males. It is possible that the old males had impaired LV relaxation, with a mild-to-moderate decrease in LV compliance, and a decline in diastolic function that was more obvious in males compared with females of the same advanced age.

LVEF and FS% are the main parameters to evaluate LV systolic function when using conventional ECG. However, conventional ECG techniques are relatively insensitive to early or subtle changes in cardiac performance, particularly in mice^19^. LV SF in mice has been assessed by M-mode echocardiography. Nonetheless, as M-mode-derived volume and mass calculations are based on measurements on a single plane, they may be susceptible to error. We found that the LV systolic function parameters (i.e., EF, IVCT, IVCT/ST, and MPI), measured via traditional ECG, were not significantly different among the age and sex groups. But after applying STE, it was found that S-GLS and S-GLSR of the old males was significantly higher than that of the young males, and S-RLS and S-RLSR showed obvious difference mainly in apex. Among the females, S-GLS and S-GLSR were similar. These values were consistent with the gold standard value (+ dp/dtmax) of LV systolic function. Therefore, LV systolic function was relatively well preserved even as age advanced in the female mice. These results are consistent with the Koch et al.^20^ study, in which there an age-dependent decrease in several systolic and diastolic function parameters was determined in the male mice, but not the females.

Arterial stiffness is an independent predictor of cardiovascular outcomes, such as myocardial infarction, cognitive decline in aging, stroke, and kidney diseases^21^. Classically, PWV can be determined from the difference (or delay) in propagation of arterial pressure waves (or flow) between two recording sites in the line of pulse travel. PWV is closely associated with the intrinsic elasticity of the arterial wall and has been considered the gold standard measure of arterial stiffness^22^. Arterial stiffening increases with age, resulting in an increase in PWV. A decrease in arterial elasticity, and thus an increase of stiffness of the central elastic arteries such as the aorta, is a powerful independent predictor of cardiovascular diseases, mortality, and morbidity^23^. Our data showed that male mice had progressive, age-related declines in aortic structure and increased aortic stiffness reflected by the ∆AADR and PWV. Female mice had only age-related changes in aortic structure, and there was no significant change in aortic function. It may be that arterial stiffness in mice develops at an earlier age males than in females. The processes by which arterial stiffness increases with age are uncertain, some scholars have found that arterial stiffness was related to collagen fibrosis deposition^24^. There has been growing interest in identifying and understanding the genetics and underlying mechanisms^25^.

We acknowledge several limitations in this study. First, the population of old females was small, and to confirm these results a larger population is required. Second, the cardiovascular function of the mice was tested only in the resting state, without considering reserve function. Finally, currently 2D STE must be analyzed off-line based on a clear 2D ultrasonic image, which cannot be analyzed in real time.

## Conclusion

Our data suggest that ageing in mice leads to changes in cardiovascular structure and cardiac diastolic function, but systolic function is relatively well preserved in females. Changes in cardiac function and arterial stiffness in male mice may occur at a younger age than in females, or are more obvious. This may be because in aging female mice, changes in cardiovascular structure are more conducive to the maintenance of functions. Finally, in mice STE is not as sensitive as traditional ECG for evaluating LV diastolic function, but it is better than traditional ECG for evaluating LV systolic function.
